# Identifying potential microRNA biomarkers for colon cancer and colorectal cancer through bound nuclear norm regularization

**DOI:** 10.3389/fgene.2022.980437

**Published:** 2022-09-22

**Authors:** Shengyong Zhai, Xiaoling Li, Yan Wu, Xiaoli Shi, Binbin Ji, Chun Qiu

**Affiliations:** ^1^ Department of General Surgery, Weifang People’s Hospital, Shandong, China; ^2^ The Second Department of Oncology, Beidahuang Industry Group General Hospital, Harbin, China; ^3^ Heilongjiang Second Cancer Hospital, Harbin, China; ^4^ Geneis Beijing Co., Ltd., Beijing, China; ^5^ Department of Oncology, Hainan General Hospital, Haikou, China

**Keywords:** colon cancer, colorectal cancer, microRNA, biomarker, microRNA-disease association, bound nuclear norm regularization

## Abstract

Colon cancer and colorectal cancer are two common cancer-related deaths worldwide. Identification of potential biomarkers for the two cancers can help us to evaluate their initiation, progression and therapeutic response. In this study, we propose a new microRNA-disease association identification method, BNNRMDA, to discover potential microRNA biomarkers for the two cancers. BNNRMDA better combines disease semantic similarity and Gaussian Association Profile Kernel (GAPK) similarity, microRNA function similarity and GAPK similarity, and the bound nuclear norm regularization model. Compared to other five classical microRNA-disease association identification methods (MIDPE, MIDP, RLSMDA, GRNMF, AND LPLNS), BNNRMDA obtains the highest AUC of 0.9071, demonstrating its strong microRNA-disease association identification performance. BNNRMDA is applied to discover possible microRNA biomarkers for colon cancer and colorectal cancer. The results show that all 73 known microRNAs associated with colon cancer in the HMDD database have the highest association scores with colon cancer and are ranked as top 73. Among 137 known microRNAs associated with colorectal cancer in the HMDD database, 129 microRNAs have the highest association scores with colorectal cancer and are ranked as top 129. In addition, we predict that hsa-miR-103a could be a potential biomarker of colon cancer and hsa-mir-193b and hsa-mir-7days could be potential biomarkers of colorectal cancer.

## 1 Introduction

Cancers are seriously threatening and endangering human health (Yang et al., 2013; Liu et al., 2021; Yang et al., 2022). Colon cancer and colorectal cancer are two of leading causes of cancer-related deaths worldwide (Lee et al., 2018; Piawah and Venook, 2019). Patients with colon cancer only have a survival rate of 10% when diagnosed at late stage. More importantly, colon cancer shows a higher incidence rate in elder populations. The survival rate of patients with colon cancer is densely associated with the size, location, and stage of the tumor. Metastasis may be the leading cause of deaths for patients suffered from late-stage colon cancer. Thus, understanding the mechanisms of colon cancer could contribute to designing more strong therapeutic options (Ma et al., 2021).

Nowadays, patients with colorectal cancer show a younger trend. In the last decade, incidence rates and death rates of colorectal cancers separately increased by 22 and 13% among adults under 50 years in the United State. However, their precise aetiologic factors still remain unknown. Many evidence demonstrate that early screening of colorectal cancer can reduce their incidence and mortality. Thus, the identification of diagnosis or prognosis biomarkers can contribute to assessment of tumour initiation, progression and therapeutic response for colorectal cancer (Sampath et al., 2021).

Many researches show that numerous RNA data play important roles in the development and metastasis of various diseases including cancers and COVID-19 (Huang et al., 2017; Peng L. et al., 2020; Xu et al., 2020; Yang et al., 2020; Zhang et al., 2021; Peng L. et al., 2022; Shen et al., 2022; Tian et al., 2022). In particular, noncoding RNAs could be biomarkers to boost drug design (Liu et al., 2020; Meng et al., 2022). For example, lncRNAs and circRNAs have been used as biomarkers of cancers (Peng et al., 2021a; Peng et al., 2021b; Chen et al., 2021; Li et al., 2021; Verduci et al., 2021; Wang et al., 2021; Peng L. H. et al., 2022). MicroRNAs (miRNAs) are a class of small non-coding RNAs with 22–24 nucleotides in length (Li et al., 2018; Chen et al., 2020). MicroRNAs can bind to mRNAs of target genes to inhibit expression of these genes. In addition, a few microRNAs may suppress tumors while other microRNAs may affect the progression and metastasis of tumors.

The dysfunction of microRNAs is densely linked to the inflammation of colon cancer. For example, Ma et al. (Ma et al., 2021) found that M2 macrophage-derived exosomal miR-155-5p may have an association with the immune escape of cells in colon cancer. Pagotto et al. (Pagotto et al., 2022) observed that the miR-483 gene could have a responsive to glucose availability for colon cancer. Miao et al. (Miao et al., 2021) identified that miR-4284 could be a therapeutic target in colon cancer. Dougherty et al. (Dougherty et al., 2021) inferred that the upregulations of microRNA-143 and microRNA-145 have close linkages with colonocytes suppresses colitis and inflammation-related colon cancer. Zhang et al. (Zhang et al., 2021) suggested that microRNA-24-3p could heighten the resistance of colon cancer cell to MTX. Yue et al. (Yue et al., 2021) reported that NEDD4 could trigger colon cancer progression through microRNA-340-5p suppression. In summary, the identification of microRNAs in the blood, tissues, and faecal matter will help us use these microRNA as biomarkers in early detection of colon cancer and thus design strong targeted therapeutic strategies for inflammation-mediated colon cancer (Peng et al., 2018; Sampath et al., 2021).

More importantly, microRNAs densely link to the carcinogenic process of colorectal cancer. For example, microRNA-143-3p can limit colorectal cancer metastases (Guo et al., 2019), microRNA-375-3p can boost chemosensitivity to 5-fluorouracil through targeting thymidylate synthase in colorectal cancer (Xu et al., 2020), microRNA-451a influences colorectal cancer proliferation (Ruhl et al., 2018), and microRNA-146a can inhibit tumorigenic inflammation of colorectal cancer (Garo et al., 2021). Biomarkers are an important strategy in early screening, prognostication, survival, and treatment response prediction for cancers. Therefore, microRNAs have been explored as biomarkers in colorectal cancer (Peng LH. et al., 2020; Ogunwobi et al., 2020).

Recently, many researchers have been devoted to microRNA biomarker identification for cancer including colon cancer and colorectal cancer by computational microRNA-disease association prediction (Peng et al., 2017; Li et al., 2021). Huang et al. (Huang et al., 2021) innovatively represented microRNA-disease-type triples as a tensor and further designed a tensor decomposition model to detect new microRNA-disease associations. Li et al. (Li et al., 2021) considered that the abnormal expression of microRNAs is densely associated with the evolution and progression of human diseases and inferred disease-related microRNAs as new biomarkers through a graph auto-encoder model. Chen et al. (Chen et al., 2021) designed a deep learning model for microRNA-disease association identification based on deep belief network. Wang et al. (2022)) pretrained a stacked autoencoder to predict potential microRNA-disease associations in an unsupervised manner. These methods effectively improved microRNA biomarker identification of human complex diseases.

In this study, we design a MicroRNA-Disease Association prediction algorithm (BNNRMDA) to find potential microRNA biomarkers for colon cancer and colorectal cancer based on disease semantic similarity, microRNA functional similarity, Gaussian association profile kernel (GAPK) similarity, and the Bound Nuclear Norm Regularization model.

## 2 Materials and methods

### 2.1 Data

#### 2.1.1 Dataset

Experimentally confirmed microRNA-disease association data can be downloaded from the HMDD database provided by Li et al. (Li et al., 2014). The hierarchical structures between diseases can be downloaded from the MeSH database (https://www.nlm.nih.gov/mesh/). Experimentally supported microRNA-gene interactions can be downloaded from TarBase (Vergoulis et al., 2012), miRTarBase (Hsu et al., 2014), and miRecords (Xiao et al., 2009). We acquired microRNA-disease associations between 495 microRNAs and 378 diseases, hierarchical structures for 4,663 diseases, and 38,089 microRNA-gene interactions between 477 microRNAs and 12,422 genes. Finally, we obtained 4,791 associations between 353 microRNAs and 327 diseases after removing microRNAs without target genes and diseases without hierarchical structures.

#### 2.1.2 Disease semantic similarity

For a known disease d , it can be described as a directed acyclic graph ( DAG ) based on the MeSH descriptor: $DAG_{d}=\left(d,T_{d},E_{d}\right)$ where $T_{d}$ denotes the set of nodes that contains d and all its ancestors, and $E_{d}$ represents corresponding direct edges. Given a disease $t\in T_{d}$ , its semantic contribution to d can be defined as Eq. 1:

$$D_{d}\left(t\right)=\{\begin{array}{c} 1\;\;\;\;\;\;\;\;\;\;\;\;\;\;\;\;\;\;\;\;\;\;\;\;\;\;\;\;\;\;\;\;\;\;\;\;\;\;\;\;\;\;\;\;\;\;\;\;\;\;\;\;\;\;\;\;\;\;\;\;\;\;\;\;\;\;\;\;\;\;\;\;\;\;\;\;\;\;\;\;\;\;if\;t\;\neq d\;\;\;\\ max\left\{\Delta *D_{d}\left(t\prime \right)\text{|}t\prime \in children\;of\;t\right\}\;\;\;\;\;\;\;\;\;\;\;\;\;\;\;\;if\;t\;\neq d\;\;\; \end{array}$$ (1)

where Δ denotes the semantic contribution decay factor ( Δ=0.5 ) (Wang et al., 2010). In general, two diseases $d_{i}$ and $d_{j}$ are more similar when they share more common ancestors. Thus, pairwise semantic similarity between $d_{i}$ and $d_{j}$ can be defined as Eq. 2:

$$S^{d}\left(d_{i},d_{j}\right)=\frac{\sum_{t\in T_{d_{i}}\cap T_{d_{j}}}\left(D_{d_{i}}\left(t\right)+D_{d_{j}}\left(t\right)\right)}{\sum_{t\in T_{d_{i}}}D_{d_{i}}\left(t\right)+\sum_{t\in T_{d_{j}}}D_{d_{j}}\left(t\right)}$$ (2)

#### 2.1.3 MicroRNA functional similarity

MicroRNA similarity can be computed based on microRNA-gene associations and gene functional network. First, the associated log-likelihood scores $LLS\left(g_{i},g_{j}\right)$ between two genes $g_{i}$ and $g_{j}$ can be calculated using HumanNet (Lee et al., 2011).

Second, $LLS\left(g_{i},g_{j}\right)$ is normalized by Eq. 3:

$$LLS_{N}\left(g_{i},g_{j}\right)=\frac{LLS\left(g_{i},g_{j}\right)-LLS_{min}}{LLS_{max}-LLS_{min}}$$ (3)

where $LLS_{min}$ and $LLS_{max}$ represent the minimum and maximum associated log-likelihood scores computed by HumanNet, respectively.

Third, similarity between $g_{i}$ and $g_{j}$ can be calculated by Eq. 4:

$$S^{g}\left(g_{i},g_{j}\right)=\{\begin{array}{c} 1\;\;\;\;\;\;\;\;\;\;\;\;\;\;\;\;\;\;\;\;\;\;\;\;\;\;\;\;\;\;\;\;\;\;\;\;\;\;\;\;\;\;\;\;\;\;\;g_{i}=g_{j}\;\;\;\;\;\;\;\;\;\;\;\;\;\;\;\;\;\;\;\;\;\;\;\;\\ 0\;\;\;\;\;\;\;\;\;\;\;\;\;\;\;\;\;\;\;\;\;\;\;\;\;\;\;\;\;\;\;\;\;\;\;\;\;\;\;\;\;e\left(g_{i},g_{j}\right)\notin HumanNet\\ LLS_{N}\left(g_{i},g_{j}\right)\;\;\;\;\;\;\;\;\;\;\;\;\;\;\;\;\;\;\;e\left(g_{i},g_{j}\right)\in HumanNet \end{array}$$ (4)

where $e\left(g_{i},g_{j}\right)$ indicates interaction between $g_{i}$ and $g_{j}$ .

Finally, the functional similarity between two microRNAs $m_{i}$ and $m_{j}$ can be computed by Eq. 5 based on their associated genes:

$$S^{m}\left(m_{i},m_{j}\right)=\frac{\sum_{g\in G_{i}}S\left(g,G_{j}\right)+\sum_{g\in G_{j}}S\left(g,G_{i}\right)}{\left|G_{i}\right|+\left|G_{j}\right|}$$ (5)

where $G_{i}$ and $G_{j}$ denotes the gene sets associated with $m_{i}$ and $m_{j}$ , respectively, $\left|G_{i}\right|$ and $\left|G_{j}\right|$ denote corresponding cardinalities, respectively, and $S\left(g,G\right)=max_{g_{i}\in G}\left\{S^{g}\left(g,g_{i}\right)\right\}$ .

#### 2.1.4 GAPK similarity

For a known disease $d_{i}$ in a microRNA-disease association matrix $X_{a\times b}$ , let the i th row of X denotes its Gaussian association profile $GAP\left(d_{i}\right)$ to represent its association features with all diseases. GAPK similarity between diseases $d_{i}$ and $d_{j}$ can be measured by Eq. 6.

$$\begin{array}{c} G_{D}\left(d_{i},d_{j}\right)=\exp\left(-\gamma_{d}\|GAP\left(d_{i}\right)-GAP{\left(d_{j}\right)\|}^{2}\right)\\ \gamma_{d}=\gamma \prime_{d}/\left(\frac{1}{a}\overset{a}{\underset{k=1}{\sum }}\mathrm{\|G}AP{\left(d_{k}\right)\|}^{2}\right) \end{array}\;$$ (6)

where $\gamma_{d}$ indicates normalized kernel bandwidth according to parameter $\gamma \prime_{d}$ , and a indicates the number of diseases.

Similarly, for a known microRNA $m_{i}$ , let the i th column of X denotes its Gaussian association profile $GAP\left(m_{i}\right)$ to describe its association features with all microRNAs. GAPK similarity between microRNAs $m_{i}$ and $m_{j}$ can be measured by Eq. 7:

$$\begin{array}{c} G_{M}\left(m_{i},m_{j}\right)=\exp\left(-\gamma_{m}\mathrm{\|G}AP\left(m_{i}\right)-GAP{\left(m_{j}\right)\|}^{2}\right)\\ \gamma_{m}=\gamma \prime_{m}/\left(\frac{1}{b}\overset{b}{\underset{k=1}{\sum }}\mathrm{\|G}AP{\left(m_{k}\right)\|}^{2}\right) \end{array}$$ (7)

where $\gamma_{m}$ indicates normalized kernel bandwidth according to parameter $\gamma \prime_{m}$ , and b indicates the number of microRNAs.

#### 2.1.5 Similarity fusion

Disease semantic similarity $S^{d}$ and GAPK similarity $G_{d}$ are fused to calculate the final disease similarity matrix $S_{D}$ by Eq. 8:

$$S_{D}=wG_{D}+\left(1-w\right)S^{d}$$ (8)

where the parameter w is applied to measure the weight between disease semantic similarity and GAPK similarity.

MicroRNA functional similarity $S^{m}$ and GAPK similarity $G_{m}$ are fused to calculate the final microRNA similarity matrix by Eq. 9:

$$S_{M}=wG_{M}+\left(1-w\right)S^{m}$$ (9)

where the parameter w is applied to measure the weight between microRNA functional similarity and GAPK similarity.

### 2.2 Heterogeneous microRNA-disease network construction

A heterogeneous microRNA-disease network is created by fusing microRNA similarity network, disease similarity network, and microRNA-disease association network. Each edge in similarity network is weighted based on the computed similarity. The heterogeneous microRNA-disease network can be described using a bipartite graph G(M,D,E) , where M and D separately represent microRNA set and disease set, $E\left(G\right)=\left\{e_{ij}\right\}\subseteq M\times D$ represents the microRNA-disease edge set. The adjacency matrix of G(M,D,E) is described as Eq. 10.

$$W=\left[\begin{array}{cc} W_{mm} & W_{md}\\ \;W_{md}^{T} & W_{dd} \end{array}\right]$$ (10)

where $W_{md}$ denotes known microRNA-disease association matrix, $W_{mm}$ and $W_{dd}$ denotes the adjacency matrices about microRNA similarity network and disease similarity network, respectively. Hence, the adjacency matrix can be rewritten as Eq. 11.

$$W=\left[\begin{array}{cc} S_{M} & X_{md}\\ \;X_{md}^{T} & S_{D} \end{array}\right]$$ (11)

### 2.3 BNNRMDA model

In known microRNA-disease association dataset, majority of microRNA-disease pairs are unknown-associated. Inspired by the bound nuclear norm regularization model provided by Yang et al. (Yang et al., 2019), in this study, we design the bounded nuclear norm regularization-based MDA prediction method to score each unknown microRNA-disease pair. We describe microRNA-disease association inference as a matrix completion problem and construct model (12) to predict new microRNA-disease associations in microRNA-disease association matrix:

$$\begin{array}{c} \underset{Y}{\min}rank\left(Y\right)\\ {\text{ subject to Ρ}}_{\text{Ω}}\left(Y\right)={\text{Ρ}}_{\text{Ω}}\left(W\right) \end{array}\;$$ (12)

where Y denotes a matrix need to complete, rank(Y) denotes the rank of Y , $W\in {ℛ}^{\left(m+n\right)\times \left(m+n\right)}$ denotes a known microRNA-disease association matrix, Ω denotes a set containing all index pairs (i,j) that correspond to known microRNA-disease associations in W , and ${\text{Ρ}}_{\text{Ω}}$ represents a projection operator on Ω by Eq. 13:

$${\left({\text{Ρ}}_{\text{Ω}}\left(Y\right)\right)}_{ij}=\{\begin{array}{c} Y_{ij},\;\;\;\;\left(i,j\right)\in \text{Ω}\\ \;\;\;\;0,\;\;\;\;\left(i,j\right)\notin \text{Ω} \end{array}\;$$ (13)

Model (12) is a non-convex model and difficult to solve. Thus, we transform it to a nuclear norm model through the nuclear norm optimization method proposed by Candes et al. (2013) by Eq. 14:

$$\begin{array}{c} \underset{A}{\min}\;\mathrm{\|Y}{\|}_{*}\\ {\text{subject to Ρ}}_{\text{Ω}}\left(Y\right)={\text{Ρ}}_{\text{Ω}}\left(W\right) \end{array}\;$$ (14)

where $Y_{*}$ represents the nuclear norm of Y .

Because the value of each element in microRNA and disease similarity matrices $S_{m}$ and $S_{d}$ is in the range of [0,1] and the value of each element in microRNA-disease association matrix $X_{md}$ is 1 or 0, the computed microRNA-disease association scores are restricted to [0,1]. Higher score indicates bigger association probability for one microRNA-disease pair. But the elements in Y are in the range of (−∞,+∞) . Therefore, we add a bounded constraint to Eq. 14 to make the computed scores in [0, 1]. Considering the affect of data noise on the prediction performance, in addition, we develop a rank minimization-based matrix completion model by Eq. 15:

$$\begin{array}{c} \underset{A}{\min}\mathrm{\|Y}{\|}_{*}\\ \text{subject to}\;\|{\text{P}}_{\text{Ω}}\left(Y\right)-{\text{Ρ}}_{\text{Ω}}{\left(W\right)\|}_{F}\leq \epsilon \end{array}\;$$ (15)

where ${\left\|.\right\|}_{F}$ indicates Frobenius norm and ϵ represents the noise level.

We introduce a soft regularization term to tolerate data noise considering the difficulty in selecting an appropriate parameter in Eq. 15. Consequently, a bound nuclear norm regularization model is built to infer potential microRNA-disease associations by Eq. 16:

$$\begin{array}{c} \underset{Y}{\min}\mathrm{\|Y}{\|}_{*}+\frac{\alpha }{2}\|{\text{Ρ}}_{\text{Ω}}\left(Y\right)-{\text{Ρ}}_{\text{Ω}}{\left(W\right)\|}_{F}^{2}\\ \text{subject to}\;\;0\leq Y\leq 1 \end{array}$$ (16)

where the parameter α is applied to weigh the importance between the nuclear norm and the error term.

Consequently, we introduce an auxiliary matrix Z and define model 17) to optimize model (16):

$$\begin{array}{c} \underset{Y}{\min}\mathrm{\|Y}{\|}_{*}+\frac{\alpha }{2}\|{\text{Ρ}}_{\text{Ω}}\left(Z\right)-{\text{Ρ}}_{\text{Ω}}{\left(W\right)\|}_{F}^{2}\\ \text{subject to}\;\;Y=Z\\ 0\leq W\leq 1 \end{array}$$ (17)

where $Y_{1}={\text{Ρ}}_{\text{Ω}}\left(W\right)$ .

Thus, the corresponding augmented Lagrange function is written as Eq. 18:

$$L\left(Z,Y,L,\alpha ,\beta \right)=\mathrm{\|Y}{\|}_{*}+\frac{\alpha }{2}\|{\text{Ρ}}_{\text{Ω}}\left(Z\right)-{\text{Ρ}}_{\text{Ω}}{\left(W\right)\|}_{F}^{2}+T_{r}\left(L^{T}\left(Y-Z\right)\right)+\frac{\beta }{2}\|Y-{\mathrm{Z\|}}_{F}^{2}$$ (18)

where L and β represent the Lagrange multiplier and penalty parameter, respectively.

At the t -th iteration, we alternatively compute one of $Y_{k+1}$ , $Z_{k+1}$ and $L_{k+1}$ by fixing other two values according to the solution from Yang et al. (Yang et al., 2019). Finally, microRNA-disease association matrix $Z_{md}^{*}$ is updated through completing the unlabeled elements in $Z_{md}$ .

## 3 Experiments

### 3.1 Experimental settings and evaluation

In this study, we perform five-fold cross validation for 10 times to investigate the microRNA-disease association inference ability of BNNRMDA. During five-fold cross validation, 80% of elements in microRNA-disease association matrix X are randomly chosen as the training set and the remaining are taken as the test set. Parameters α , β ,  w , and γ′ are set by grid search. We find that BNNRMDA obtain the best AUC when the four parameters are set as α=1 , β=10 ,  w=0.3 , and γ′=0.5 , respectively. Therefore, we set the four parameters as corresponding values. In addition, AUC is widely used to measure the performance of association prediction methods, and thus we use it to measure the performance of BNNRMDA.

### 3.2 Performance measurement

To measure the microRNA-disease association prediction performance of BNNRMDA, we compare it with MIDPE (Xuan et al., 2015), MIDP (Xuan et al., 2015), RLSMDA (Chen and Yan, 2014), GRNMF (Xiao et al., 2018), and LPLNS (Li et al., 2018). MIDP (Xuan et al., 2015) and MIDPE (Xuan et al., 2015) are two random walk-based microRNA-disease association prediction methods. MIDP is used to detect association information for microRNAs related to diseases. MIDPE is used to detect association information through the bilayer network. RLSMDA (Chen and Yan, 2014) is a semi-supervised learning-based microRNA-disease association inference framework. GRNMF (Xiao et al., 2018) is a graph regularized non-negative matrix factorization-based microRNA-disease association prediction model. In addition, GRNMF built an association probability profile for each disease or miRNA based on a weighted nearest K neighbor profiles. LPLNS (Li et al., 2018) combined label propagation and linear neighborhood similarity for microRNA-disease association prediction. MIDP, MIDPE, RLSMDA, GRNMF, and LPLNS obtained better AUCs for microRNA-disease association prediction. Table 1 shows the AUC values of six microRNA-disease association prediction methods under cross validation.

**TABLE 1:** AUCs of microRNA-disease association prediction methods under cross validation.

Method	MIDPE	MIDP	RLSMDA	GRNMF	LPLNS	BNNRMDA
AUC	0.7820	0.8256	0.8555	0.8963	0.9034	0.9071

From Table 1, we can find that BNNRMDA obtains better AUC of 0.9071 than MIDPE, MIDP, RLSMDA, GRNMF, and LPLNS. Compared to MIDPE, MIDP, RLSMDA, GRNMF, and LPLNS, BNNRMDA increases the performance of 13.79, 8.98, 5.69, 1.19, and 0.41% based on the AUC value, respectively. The results show that our proposed BNNRMDA method can effectively predict new microRNA-disease associations.

### 3.3 Case study

In the above section, we have computed the performance of BNNRMDA. The results show that BNNRMDA obtains better AUC and outperforms other five microRNA-disease association prediction methods. We continue to implement case analyses to identify possible microRNA biomarkers for colon cancer and colorectal cancer.

#### 3.3.1 Inferring possible microRNA biomarkers for colon cancer

**FIGURE 1:**
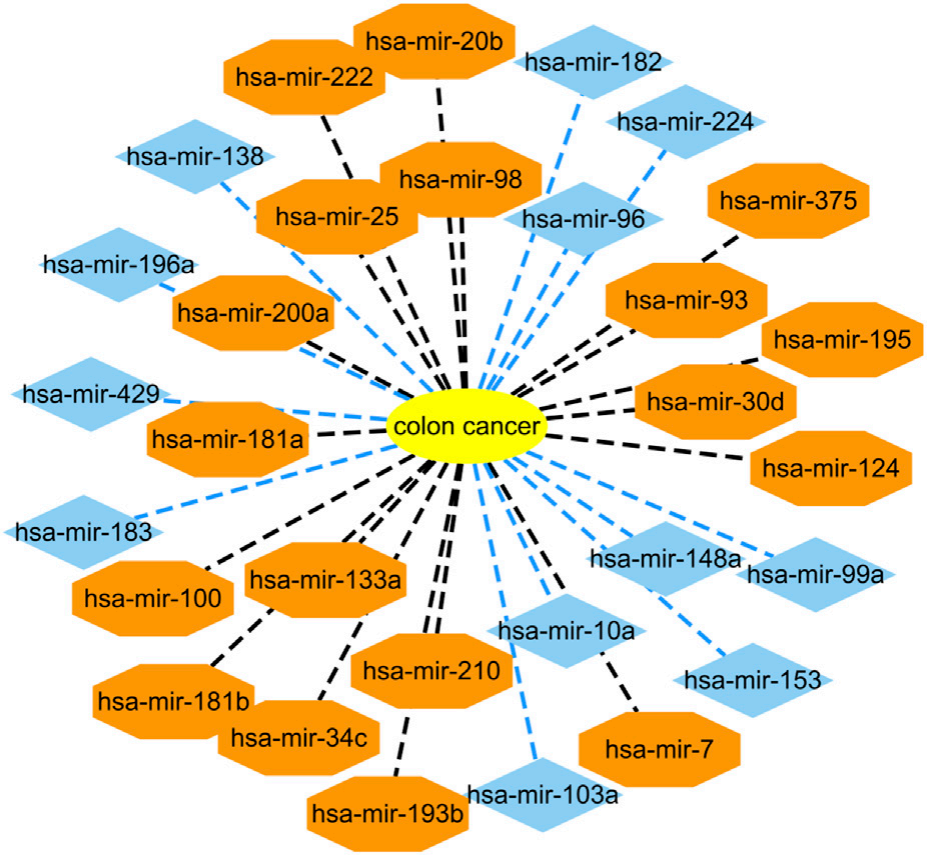
Associations between the predicted top 30 microRNAs and colon cancer except for known 73 microRNA-colon cancer associations in the HMDD database that are predicted to have the highest association scores with colon cancer. Black dot lines denote associations between microRNAs and colon cancer and these associations have been reported by publications. Blue dot lines denote associations between microRNAs and colon cancer and these associations are unknown and need to experimental validation.

Colon cancer is a common malignant tumor and has a very high incidence rate in adult with age of 40–50 (Zhu et al., 2020; Liu et al., 2021). More importantly, it has no any symptoms in the early stage. Therefore, it is important to infer possible biomarkers to boost the diagnosis and treatment for colon cancer (Liu et al., 2021). Among the HMDD dataset, there are 73 known microRNAs associated with colon cancer among 353 microRNAs. Based on the proposed BNNRMDA method, we compute the association score for each microRNA-disease pair. The results show that all 73 known microRNAs associated with colon cancer in the HMDD database have the highest association scores with colon cancer and are ranked as top 73. We continue to investigate the following 30 miRNAs that have higher association scores with colon cancer and are ranked as 74–103. The results are shown in Table 2 and Figure 1. From Table 2 and Figure 1, we can find that 18 microRNAs are confirmed to associate with colon cancer by literature retrieval. In addition, 12 microRNAs are inferred to associate with colon cancer and are potential biomarkers of colon cancer.

**TABLE 2:** The inferred top 30 microRNAs associated with colon cancer except for 73 known microRNAs.

Rank	MicroRNA	Evidence	Rank	MicroRNA	Evidence
1	hsa-mir-200a	25371200	16	hsa-mir-99a	Unconfirmed
2	hsa-mir-375	29930763	17	hsa-mir-195	26064276
3	hsa-mir-222	27855613	18	hsa-mir-96	Unconfirmed
4	hsa-mir-30d	28651493	19	hsa-mir-148a	Unconfirmed
5	hsa-mir-103a	Unconfirmed	20	hsa-mir-98	28025745
6	hsa-mir-100	28032929	21	hsa-mir-34c	https://doi.org/10.1166/jbt. 2018.1859
7	hsa-mir-181a	25977338	22	hsa-mir-182	Unconfirmed
8	hsa-mir-133a	29930763	23	hsa-mir-20b	33044899
9	hsa-mir-429	Unconfirmed	24	hsa-mir-124	30980700
10	hsa-mir-224	Unconfirmed	25	hsa-mir-7	26648422
11	hsa-mir-93	22180714	26	hsa-mir-193b	31007734
12	hsa-mir-25	23435373	27	hsa-mir-210	27611932
13	hsa-mir-181b	18172508	28	hsa-mir-10a	Unconfirmed
14	hsa-mir-183	Unconfirmed	29	hsa-mir-138	Unconfirmed
15	hsa-mir-153	Unconfirmed	30	hsa-mir-196a	Unconfirmed

In addition, we infer that microRNA hsa-mir-103a may associate with colon cancer. Wnt signaling pathway is hyper-activated in many human cancers. Therefore, Wnt pathway demonstrates promising diagnostic and therapeutic effect in cancer medicine. Fasihi et al. (2018) found that hsa-miR-103a may be a possible regulator of Wnt signaling pathway by detecting its effect on Wnt pathway components in colorectal cancer-originated cell lines and its expression in colorectal cancer tissues. They also found that hsa-miR-103a has an upregulation function in colorectal cancer tissues through RT-qPCR and its overexpression could cause elevated Wnt activity. Therefore, we infer that hsa-miR-103a could be a potential biomarker of colon cancer (Fasihi et al., 2017).

#### 3.3.2 Inferring possible microRNA biomarkers for colorectal cancer

Colorectal cancer is the third leading cause of cancer-related deaths in the United States. In the United State, there are about 1.85 million cases and 850 thousand deaths annually. In 2020, there are 53,200 colorectal cancer deaths in the United State. Among new colorectal cancer diagnoses, approximately 20% of patients suffered from metastatic disease and approximately 25% of patients suffered from localized disease that may later develop metastases. Of patients who are diagnosed as metastatic colorectal cancer, about 70–75% of patients survive more than 1 year, about 30–35% patients survive more than 3 years, and less than 20% patients survive more than 5 years (Xie et al., 2020; Biller and Schrag, 2021).

**FIGURE 2:**
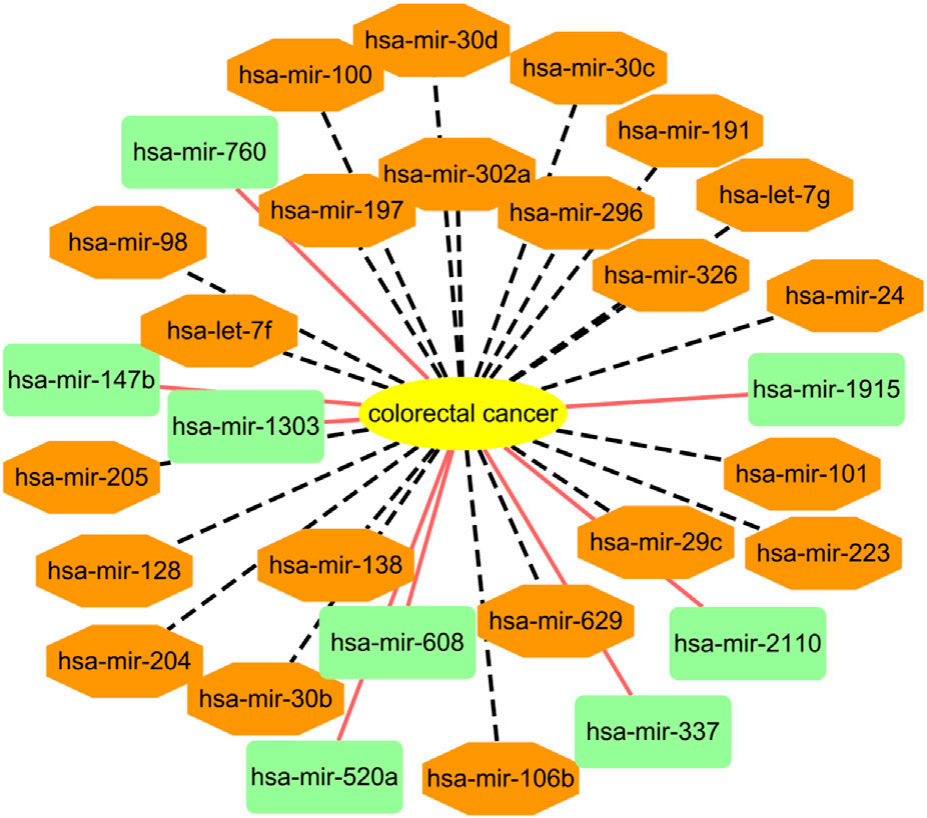
Associations between the predicted top 30 microRNAs and colorectal cancer except for known 129 microRNA-colorectal cancer associations in the HMDD database. Black dot lines denote associations between microRNAs and colorectal cancer and these associations have been reported by publications. Orange solid lines denote associations between microRNAs and colorectal cancer and these associations are unknown and need to experimental validation.

Among the HMDD dataset, there are 137 known microRNAs associated with colorectal cancer among 353 microRNAs. Based on the proposed BNNRMDA method, we compute the association score for each microRNA-colorectal cancer pair. The results show that 129 known microRNAs associated with colorectal cancer in the HMDD database have the highest association scores with colorectal cancer and are ranked as top 129. We continue to investigate the following 30 miRNAs that have higher association scores with colorectal cancer and are ranked as 130–159. The results are shown in Table 3 and Figure 2. From Table 3 and Figure 2, we can find that 8 microRNAs are known to associate with colorectal cancer in the HMDD database. In addition, the remaining 22 microRNAs are inferred to associate with colorectal cancer and are reported by publications. The results confirm the strong microRNA identification performance of BNNRMDA for colorectal cancer. In addition, we predict that hsa-mir-193b and hsa-mir-7 days may associate with colorectal cancer and need validation.

**TABLE 3:** The inferred top 30 microRNAs associated with colorectal cancer except for 129 known microRNAs.

Rank	MicroRNA	Evidence	Rank	MicroRNA	Evidence
1	hsa-mir-191	18079988	16	hsa-mir-223	27759076
2	hsa-mir-760	the HMDD database	17	hsa-mir-100	25973296
3	hsa-mir-337	the HMDD database	18	hsa-mir-204	25209181
4	hsa-mir-1915	the HMDD database	19	hsa-let-7g	18172508
5	hsa-mir-24	30375302	20	hsa-mir-106b	34070923
6	hsa-mir-520a	the HMDD database	21	hsa-mir-296	28209128
7	hsa-mir-101	30797148	22	hsa-let-7f	29805607
8	hsa-mir-138	27248318	23	hsa-mir-29c	29262657
9	hsa-mir-608	the HMDD database	24	hsa-mir-30c	25799050
10	hsa-mir-1303	the HMDD database	25	hsa-mir-30b	32112903
11	hsa-mir-629	30042169	26	hsa-mir-302a	31754405
12	hsa-mir-2110	the HMDD database	27	hsa-mir-326	25760058
13	hsa-mir-147b	the HMDD database	28	hsa-mir-98	34370878
14	hsa-mir-205	29488611	29	hsa-mir-128	30257253
15	hsa-mir-197	30106114	30	hsa-mir-30d	28651493

## 4 Conclusion

Colon cancer and colorectal cancer are two of leading causes of cancer-related deaths worldwide and are seriously threatening human health. Inference of diagnosis or prognosis biomarkers for colon cancer and colorectal cancer can help to evaluate their initiation, progression and therapeutic response. In this study, we developed a new microRNA-disease association prediction method, BNNRMDA, to find possible microRNA biomarkers for colon cancer and colorectal cancer. BNNRMDA effectively integrated disease semantic similarity and GAPK similarity, microRNA function similarity and GAPK similarity, and bound nuclear norm regularization.

Compared to other five classical microRNA-disease association prediction methods, BNNRMDA obtains the best AUC of 0.9071, demonstrating its powerful microRNA-disease association prediction performance. We continue to use the proposed BNNRMDA method for finding possible microRNA biomarkers for colon cancer and colorectal cancer. The results show that hsa-miR-103a could be a potential biomarker of colon cancer and hsa-mir-193b and hsa-mir-7 days could be potential biomarkers of colorectal cancer.

Our proposed BNNRMDA method fully considers the affect of Gaussian association profile similarity on the prediction performance. In addition, the bound nuclear norm regularization approach can effectively learn the intrinsic distribution of data. Therefore, BNNRMDA significantly outperform other MDA prediction methods. Although BNNRMDA obtains better AUC, its performance including AUC, precision, recall, and accuracy need to further improve. In the future, we will improve the bound nuclear norm regularization model to discover possible biomarkers for colon cancer and colorectal cancer.

## Data Availability

The original contributions presented in the study are included in the article/supplementary material, further inquiries can be directed to the corresponding author.
